# Adolescent and adult perceptions of the effects of larger size graphic health warnings on conventional and plain tobacco packs in India: A community-based cross-sectional study

**DOI:** 10.18332/tid/110677

**Published:** 2019-10-01

**Authors:** Gaurang P. Nazar, Monika Arora, Vinay K. Gupta, Tina Rawal, Amit Yadav, Nanda K. Kannuri, Surbhi Shrivastava, Nathan Grills, Premila Webster

**Affiliations:** 1 Health Promotion Division, Public Health Foundation of India, Gurugram, India; 2 HRIDAY, New Delhi, India; 3 Center for Tobacco Control Research and Education, University of California, San Francisco, United States; 4 Indian Institute of Public Health, Hyderabad, India; 5 Nossal Institute for Global Health, University of Melbourne, Melbourne, Australia; 6 Nuffield Department of Population Health, University of Oxford, Oxford, United Kingdom

**Keywords:** perception, tobacco, plain packaging, graphic health warnings, India

## Abstract

**INTRODUCTION:**

We studied adolescent and adult perceptions of the effects of larger size, 85% versus 40%, Graphic Health Warnings (GHWs) on conventional and plain tobacco packs, in India.

**METHODS:**

A cross-sectional survey was conducted with 2121 participants (aged ≥13 years), during the period 2015–16, in Delhi and Telangana, India. Four categories of GHWs on tobacco packs were shown: A – 40% existing (April 2013–April 2016), B – 40% new (April 2016–present), C – 85% new, and D – plain packs (85% new). Regression models tested percentage differences in choice of categories for eight outcomes, adjusted for gender, area of residence, socioeconomic status, age, and tobacco use.

**RESULTS:**

Of the total 2121 participants, 1120 were from Delhi, 1001 from Telangana, 50% were males, 62% were urban residents, 12% were adolescents, and 72% had never used tobacco. Among packs shown, the majority of participants perceived the 85% size GHWs more effective than the 40% size GHWs across all outcomes. The perceived increase in noticeability of GHWs was 45% for category C (p<0.05) and 43.5% for category D (p<0.05) versus category B. In Delhi, participants perceived plain packs to be most effective in motivating quitting, preventing initiation and conveying the health message. In Telangana, adolescents believed GHWs on plain packs were most noticeable, most effective for quitting and preventing initiation.

**CONCLUSIONS:**

The larger size 85% GHWs were perceived to be more effective in increasing noticeability of warnings, motivating cessation, preventing initiation, and conveying the intended health message. Support for plain packaging was higher in Delhi and among adolescents in Telangana.

## INTRODUCTION

Tobacco use leads to more than 1.3 million deaths annually in India^1,2^, and this is expected to rise to 1.5 million by 2020^3^. Adolescents are particularly vulnerable to tobacco industry advertising and promotion, which can lead to initiation of tobacco use^4^. The mean age of initiation of tobacco in India is 18.9 years^1,2^. Therefore, adolescents represent an important target for tobacco prevention and control efforts. Tobacco control in India is complicated by the range of tobacco products and packs available. The Government of India (GoI) implemented the Cigarettes and Other Tobacco Products Act (COTPA) in 2003^5^. Section 7 of COTPA mandates the display of graphic health warnings (GHWs) on all tobacco packs^5,6^. GHWs covering 40% of the principal display area (PDA) [front panel] of the pack were introduced in India from 31 May 2009, and were rotated in the years 2011, 2013 and 2016^7^. In 2014, the GoI notified for larger, field-tested GHWs (covering top 85% of the PDA [front and back] of tobacco packs), which were implemented from April 2016^6,8^. The Karnataka High Court struck down the 2014 notification in December 2017 but the notification was upheld by the Supreme Court of India in January 2018^9-11^. However, legal challenges continue to threaten the implementation of large GHWs in India.

The implementation of plain packaging with large GHWs is the next step towards a comprehensive tobacco control policy, and specifically, to prevent tobacco use among adolescents. Plain packaging of tobacco products was implemented as a demand-reduction strategy to protect young people initially in Australia in 2012^12^, followed by France, the United Kingdom, New Zealand, Norway, and Ireland^12^. Australian research suggests that plain packaging has accelerated the decline in smoking prevalence, reduced the appeal of tobacco packs as well as the ability of packs to mislead consumers, and enhanced the effectiveness of GHWs^13^. Plain packaging in India would also be expected to make tobacco packs less attractive. At present, COTPA (2003)^5^ allows on-pack advertising, which makes tobacco packs attractive. An India–Australia High-level Taskforce was set up in India to explore the feasibility of plain packaging in 2012. The preliminary research evidence^14,15^ led to the introduction of a private members’ bill on plain packaging in the Indian Parliament^16^.

In countries that have implemented plain packaging, public consultations were undertaken to obtain the views of the public and experts on the concept of plain packaging^17^. Such public consultations and research on perceptions of the impact of plain tobacco packaging and larger GHWs (from 40% to 85%) on various tobacco-use outcomes have not been undertaken in India.

The objective of this study was to assess perceptions of Indian adolescents (aged 13–17 years) and adults (≥18 years) of the effect of the 2016 GHWs and their increased size from 40% to 85% on conventional tobacco packs compared with plain packs.

The pre-specified hypothesis was that increasing the size of GHWs to 85% and placing them on plain packaging would increase the noticeability of the warnings by at least 5% compared with 40% size warnings placed on conventional packs.

## METHODS

### Study design

**Figure 1:**
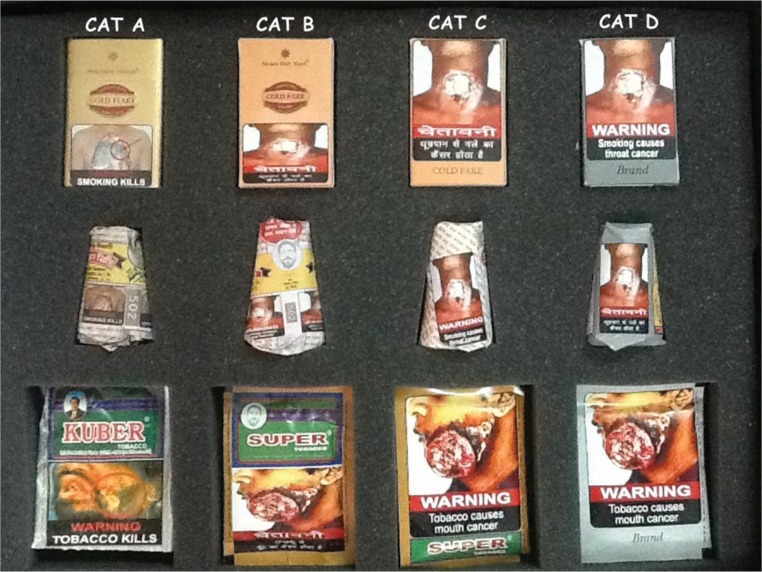
Four categories of tobacco product packs shown to the participants

This community-based, cross-sectional study was conducted in February–March 2016, using an interviewer-administered questionnaire during individual face-to-face interviews with study participants. The study was conducted after the notification (October 2014) but before the implementation (April 2016) of new 85% sized GHWs in India. Hence, the 40% size GHWs that were in force between April 2013 and April 2016 were imprinted on the tobacco packs when this study was conducted. Therefore, these packs were included in this study. Conventional tobacco packs with 40% sized existing (April 2013 – April 2016) warnings (pack category A), dummy tobacco packs with 40% new (April 2016 – present) warnings (pack category B), dummy tobacco packs with 85% new warnings on conventional packs (pack category C) and dummy tobacco packs with 85% new warnings on plain packs (pack category D) were shown (Figure 1) to participants to elicit responses to questions asked by the interviewer using the questionnaire.

### Study setting

The study was conducted in communities of New Delhi (urban) and Najafgarh (rural) districts in the National Capital Territory (NCT) of Delhi, and in Hyderabad (urban) and Ranga Reddy (rural) districts in the state of Telangana. The NCT of Delhi represented north India and the state of Telangana represented south India.

### Participants

The sample size was estimated for the difference between two proportions, based on the reported noticeability of GHWs on tobacco packs as the outcome, which was considered to be 28% based on a previous Indian study^15^. Assuming a 5% increase in the proportion of the population that will notice (reportedly observe) warnings after increasing the size from 40% to 85%, and placing these on plain packs, the minimum estimated sample size was 1326. The sample size was finalised as 2000 (1000 in each state). The sample was further divided between rural and urban areas and age groups based on 2011 census population ratio^18^.

Participants aged ≥13 years were selected, consistent with the nationally representative surveys: Global Youth Tobacco Survey (GYTS)^19^, and Global Adult Tobacco Survey (GATS)^1^. Also, consistent with the sampling method used by GYTS and GATS, a two-stage sampling scheme was used. The primary sampling units (PSU) were villages in the rural area and wards in urban areas. These were selected using probability proportional to size. Twenty households from each PSU and one participant from each household were selected. As no accurate household lists were available in villages, each village was identified on Google maps and geographically divided into two equal parts. From each part, 10 males and 10 females were selected. This was based on the Expanded Program on Immunization method used by WHO and UNICEF in the absence of suitable sampling frames^20^. Similarly, wards in the urban area were divided into two parts and 10 houses were selected from each part through a systematic sampling technique. The overall response rate was 70%.

### The questionnaire

The questionnaire was developed in consultation with experts in tobacco control in India and Australia, building on the previous feasibility study conducted by the India–Australia High-level Taskforce^15^. It included components from previously validated tools used for testing perceptions of the effects of modified packaging with different sizes of GHWs in Australia^21^, and in Canada with adults^22^, and young people^23^. Data were collected on demography, tobacco use behaviour, perceived noticeability of GHWs, the appeal of tobacco packs, and perceived effectiveness of GHWs on various indicators related to tobacco use. Participants were shown four categories of tobacco packs simultaneously^21^ to elicit their responses during the survey (Figure 1).

The questionnaire was developed in English, translated to Hindi (Delhi) and Telugu (Telangana) and then back-translated to English. After piloting the questionnaire with 100 participants in Delhi and Hyderabad to test face and content validity, appropriate changes were incorporated. Written informed consent was obtained from all adult participants and informed assent was obtained from adolescents, after obtaining their parents’/guardians’ consent. Ethics approval for the study was obtained from the Institutional Ethics Committee at Public Health Foundation of India (TRC-IEC-252/15).

### Outcome variables

The outcome variables were participant perceptions of the four categories of tobacco product packs shown to them. Participants were asked which of the four categories they thought would:

- Be most effective in motivating tobacco users to quit;
- Be most effective in preventing initiation of tobacco products among non-users;
- Be most likely to make participants think that the health risks of tobacco use are extremely serious;
- Make GHWs stand out the most/make them most noticeable;
- Be most effective in conveying the GHW message so that it is easily understood;
- Be most likely to attract an adult to use the product;
- Be most likely to attract children and adolescents to use the product.

Support for GoI-introduced larger GHW, from 40% to 85%, was also assessed.

### Data analysis

The socioeconomic status (SES) variable combined education, occupation and income through Kuppuswamy’s SES scale criteria^24^. Tobacco use status was categorized as user (current- and ever-user) and non-user. Age groups were dichotomized as adolescents (aged 13–17 years) and adults (aged ≥18 years). Stata v·13·1 (StataCorp LP, TX) was used for data analyses. Frequency distributions and percentages were provided for sociodemographic characteristics and responses about perceptions of the four categories of tobacco packs. Associations of perceptions between age groups, area (rural/urban), gender, SES groups and tobacco use status were obtained using Pearson’s chi-squared test or Fisher’s exact test, as appropriate (Supplementary Tables S2–S6). We used linear regression to test the differences in percentages between the choice of tobacco packs (C and B; D and B; D and C) for each outcome, after adjusting for covariates significantly associated with the outcomes. Comparisons with pack category A are presented in Supplementary files (Tables S7–S9) and not discussed in great detail in this paper since they became non-existent in India soon after the study and hence irrelevant for tobacco control policy.

## RESULTS

### Demographic characteristics

The study included 2121 participants (Delhi 1120, Telangana 1001) (Table 1). Fifty per cent of participants were males, while 12.5% of participants came from the rural area in Delhi compared with 66% in Telangana. About 12% of the participants were aged 13–17 years, just under 50% were aged 25–44 years and about one-fifth were aged ≥45 years. Half of the participants were in the low SES (lower and upper lower) category, 46% belonged to middle SES (lower middle and upper middle) category and only 4% belonged to upper SES category. A quarter of participants currently used tobacco while 72% had never used tobacco.

**Table 1:** Sociodemographic characteristics of respondents

	*Delhi (n=1120)*		*Telangana (n=1001)*		*Total (N=2121)*	
	*N*	*%*	*N*	*%*	*N*	*%*
**Gender**						
Male	560	50	500	50	1060	50
Female	560	50	501	50	1061	50
**Area**						
Urban	980	87.5	341	34	1321	62
Rural	140	12.5	660	66	800	38
**Age group (years)**						
13–17	147	13	100	10	247	12
18–24	183	16	207	21	390	18
25–44	528	47	501	50	1029	49
≥45	262	23	193	19	455	21
Socioeconomic status (SES)*						
Lower	16	2	49	5	65	3
Upper lower	444	40	551	55	995	47
Lower middle	376	34	254	25	630	30
Upper middle	222	20	105	11	327	16
Upper	41	4	41	4	82	4
Tobacco use status**						
Current user	252	23	277	28	529	25
Ever user	35	3	18	2	53	2.50
Never user	833	74	704	70	1537	72.5

* Based on the Kuppuswamy scale; 22 cases missing (21 in Delhi and 1 in Telangana).

** Two cases missing in Telangana.

### Participant perceptions of the effect of pack categories

Adjusted regression results suggest that the percentage of participants who perceived increased noticeability of GHWs was significantly higher for the 85% size GHWs on conventional packs (category C) as well as for 85% size GHWs on plain packs (category D), compared with 40% size GHWs on conventional packs (category B) (Table 2). The reported increase in perceived noticeability was 45% for category C compared with category B (p<0.05), and 43.5% for category D compared with category B (p<0.05). The difference in perceived noticeability was not significant between category D compared with category C. In Delhi, the reported increase in perceived noticeability was higher for category D than category C, although the difference between D and C was not significant (Table 3).

**Table 2:** Percentage difference between the choice of tobacco pack category (Overall, n=2121)

	*Between pack C and B*		*Between pack D and B*		*Between pack D and C*	
	*Unadjusted % difference ( 95% CI)*	Adjusted % difference ( 95% CI)*	*Unadjusted % difference ( 95% CI)*	Adjusted % difference ( 95% CI)*	*Unadjusted % difference ( 95% CI)*	Adjusted % difference ( 95% CI)*
1. Most effective in motivating tobacco users to quit	**49.34 (46.99 – 51.69)**	**49.38 (47.05 – 51.72)**	**40.94 (38.62 – 43.26)**	**41.03 (38.71 – 43.34)**	**-8.40 (-12.55 – -4.24)**	-8.36 (-12.50 – -4.21)
2. Most effective in preventing initiation of tobacco use	**39.17 (36.90 – 41.44)**	**39.19 (36.91 – 41.47)**	**49.98 (47.66 – 52.29)**	**50.02 (47.69 – 52.35)**	**10.81 (6.71 – 14.91)**	10.83 (6.71 – 14.96)
3. Most likely to make you think that health risks of tobacco are extremely serious	**46.22 (43.87 – 48.58)**	**46.27 (43.92 – 48.62)**	**43.91 (41.56 – 46.25)**	**43.86 (41.52 – 46.20)**	-2.31 (-6.48 – 1.86)	-2.41 (-6.58 – 1.76)
4. GHWs are most noticeable	**45.25 (42.90 – 47.61)**	**45.26 (42.91 – 47.60)**	**43.51 (41.16 – 45.86)**	**43.51 (41.16 – 45.86)**	-1.74 (-5.89 – 2.40)	-1.75 (-5.89 – 2.39)
5. Message conveyed by the GHW is easiest to understand	**50.26 (47.94 – 52.57)**	**50.26 (47.95 – 52.57)**	**41.95 (39.66 – 44.24)**	**41.95 (39.67 – 44.24)**	**-8.30 (-12.48 – -4.13)**	-8.30 (-12.47 – -4.13)
6. Most likely to lure adults into using tobacco products	**-13.46 (-15.54 – -11.37)**	**-13.21 (-15.27 – -11.14)**	**-15.67 (-17.63 – -13.72)**	**-15.45 (-17.37 – -13.53)**	**-2.22 (-3.57 – -0.87)**	-2.24 (-3.60 – -0.88)
7. Most likely to lure children and adolescents into using tobacco products	**-12.07 (-14.17 – -9.98)**	**-11.72 (-13.80 – -9.64)**	**-14.00 (-15.99 – -12.02)**	**-13.68 (-15.64 – -11.71)**	**-1.93 (-3.38 – -0.48)**	-1.95 (-3.42 – -0.49)
8. Support for pack category	**49.98 (47.55 – 52.40)**	**50.17 (47.82 – 52.52)**	**39.12 (36.75 – 41.50)**	**39.10 (36.76 – 41.44)**	**-10.85 (-15.01 – -6.70)**	**-11.07 (-15.11 – -7.03)**

* Linear regression model adjusted for covariates that were significantly associated with the outcome. The covariates were gender (female/male), area (rural/urban), socioeconomic status (low/middle/high), age groups (13–17 /18–24 /25–44 /≥45 years) and tobacco use (never user/user). Q1–SES excluded; Q2–age group excluded; Q3–gender/SES excluded; Q4–gender/SES/tobacco use excluded; Q5–SES/tobacco use/age group excluded; Q6, 7, 8–age group and tobacco use excluded. Bold numbers indicate significance (p<0.05).

**Table 3:** Percentage difference between the choice of tobacco pack category (Delhi, n=1120)

	*Between pack C and B*		*Between pack D and B*		*Between pack D and C*	
	*Unadjusted % difference ( 95% CI)*	Adjusted % difference ( 95% CI)*	*Unadjusted % difference ( 95% CI)*	Adjusted % difference ( 95% CI)*	*Unadjusted % difference ( 95% CI)*	Adjusted % difference ( 95% CI)*
1. Most effective in motivating tobacco users to quit	**37.86 (34.57 – 41.14)**	**37.86 (34.57 – 41.14)**	**47.77 (44.41 – 51.13)**	**47.77 (44.43 – 51.11)**	**9.91 (4.27 – 15.55)**	**9.91 (4.29 – 15.53)**
2. Most effective in preventing initiation of tobacco use	**37.41 (34.20 – 40.62)**	**37.21 (33.98 – 40.45)**	**45.80 (42.51 – 49.09)**	**45.80 (42.51 – 49.09)**	**8.39 (2.85 – 13.93)**	**8.55 (3.00 – 14.10)**
3. Most likely to make you think that health risks of tobacco are extremely serious	**42.41 (39.02 – 45.80)**	**42.41 (39.02 – 45.80)**	**42.05 (38.67 – 45.44)**	**42.05 (38.70 – 45.40)**	-0.36 (-6.02 – 5.31)	-0.36 (-6.02 – 5.31)
4. GHWs are most noticeable	**37.32 (33.95 – 40.69)**	**37.32 (33.95 – 40.69)**	**43.48 (40.05 – 46.91)**	**43.48 (40.05 – 46.91)**	**6.16 (0.59 – 11.73)**	6.16 (0.59 – 11.73)
5. Message conveyed by the GHW is easiest to understand	**42.89 (39.59 – 46.20)**	**42.88 (39.59 – 46.17)**	**44.59 (41.28 – 47.90)**	**44.62 (41.36 – 47.88)**	1.70 (-4.00 – 7.4)	**1.73 (-3.90 – 7.38)**
6. Most likely to lure adults into using tobacco products	**-23.61 (-26.99 – -20.23)**	**-23.31 (-26.70 – -19.92)**	**-25.22 (-28.47 – -21.97)**	-24.94 (-28.19 – -21.69)	-1.61 (-3.76 – 0.54)	-1.63 (-3.82 – 0.56)
7. Most likely to lure children and adolescents into using tobacco products	**-22.68 (-25.84 – -19.51)**	**-22.20 (-25.38 – -19.02)**	**-21.70 (-24.94 – -18.45)**	**-21.20 (-24.44 – -17.96)**	0.98 (-1.10 – 3.06)	1.00 (-1.12 – 3.12)
8. Support for pack category	**28.21 (24.80 – 31.62)**	**28.21 (24.77 – 31.64)**	**51.43 (47.79 – 55.07)**	**51.59 (47.93 – 55.26)**	23.21 (17.72 – 28.71)*	**23.38 (17.84 – 28.92)**

* Linear regression model adjusted for covariates that were significantly associated with the outcome. The covariates were gender (female/male), area (rural/urban), socioeconomic status (low/middle/high), age groups (13–17 /18–24 /25–44 /≥45 years) and tobacco use (never user/user). Q1–SES excluded; Q2–age group excluded; Q3–gender/SES excluded; Q4–gender/SES/tobacco use excluded; Q5–SES/tobacco use/age group excluded; Q6, 7, 8–age group and tobacco use excluded. Bold numbers indicate significance (p<0.05).

Category D packs were perceived to be more effective than category C in preventing initiation among non-users when each was compared with category B (50% vs 39%) (Table 2). However, the difference between categories D and C was not significant. In Delhi, category D was perceived to be significantly more effective for this outcome than category C by 8% (Table 3). Further, in Delhi, category D was perceived to be significantly more effective than category C for motivating tobacco users to quit, by almost 10%.

Category B packs compared with category C, were perceived to be more likely to lure adults as well as adolescents into using tobacco products, than category B compared with D (Table 2). However, no significant differences were observed between categories D and C for these outcomes. The results were almost consistent in Delhi and Telangana (Tables 3 and 4).

**Table 4:** Percentage difference between the choice of tobacco pack category (Telangana, n=1001)

	*Between pack C and B*		*Between pack D and B*		*Between pack D and C*	
	*Unadjusted % difference ( 95% CI)*	Adjusted % difference ( 95% CI)*	*Unadjusted % difference ( 95% CI)*	Adjusted % difference ( 95% CI)*	*Unadjusted % difference ( 95% CI)*	Adjusted % difference ( 95% CI)*
1. Most effective in motivating tobacco users to quit	**62.20 (59.01 – 65.38)**	**62.34 (59.21 – 65.47)**	**33.30 (30.19 – 36.40)**	**33.45 (30.39 – 36.52)**	**-28.90 (-34.79 – -23.01)**	-28.88 (-34.70 – -23.07)
2. Most effective in preventing initiation of tobacco use	**41.14 (37.94 – 44.34)**	**41.35 (38.18 – 44.51)**	**54.65 (51.41 – 57.89)**	**54.73 (51.52 – 57.93)**	**13.51 (7.41 – 19.61)**	13.38 (7.33 – 19.43)
3. Most likely to make you think that health risks of tobacco are extremely serious	**50.50 (47.27 – 53.73)**	**50.61 (47.40 – 53.81)**	**45.99 (42.77 – 49.21)**	**45.88 (42.66 – 49.10)**	-4.51 (-10.67 – 16.52)	-4.72 (-10.87 – 1.42)
4. GHWs are most noticeable	**54.15 (50.97 – 57.33)**	**54.15 (50.97 – 57.33)**	**43.54 (40.38 – 46.71)**	**43.54 (40.38 – 46.71)**	**-10.61 (-16.76 – -4.46)**	-10.61 (-16.75 – -4.46)
5. Message conveyed by the GHW is easiest to understand	**58.50 (55.34 – 61.66)**	**58.51 (55.35 – 61.66)**	**39.00 (35.87 – 42.13)**	**38.99 (35.87 – 42.11)**	**-19.50 (-25.56 – -13.44)**	**-19.51 (-25.56 – -13.47)**
6. Most likely to lure adults into using tobacco products	**-2.10 (-4.18 – -0.02)**	**-2.09 (-4.15 – -0.03)**	**-5.00 (-6.77 – -3.23)**	**-4.99 (-6.75 – -3.24)**	**-2.90 (-4.45 – -1.35)**	-2.91 (-4.45 – -1.37)
7. Most likely to lure children and adolescents into using tobacco products	**-0.2 (-2.68 – 2.28)**	**-0.19 (-2.66 – 2.28)**	**-5.4 (-7.41 – -3.39)**	**-5.40 (-7.40 – -3.39)**	**-5.2 (-7.19 – -3.20)**	-5.21 (-7.20 – -3.22)
8. Support for pack category	**74.37 (71.65 – 77.10)**	**74.35 (71.66 – 77.04)**	**25.32 (22.61 – 28.04)**	**25.34 (22.66 – 28.03)**	**-49.05 (-54.46 – -43.64)**	**-49.01 (-54.35 – -43.67)**

* Linear regression model adjusted for covariates that were significantly associated with the outcome. The covariates were gender (female/male), area (rural/urban), socioeconomic status (low/middle/high), age groups (13–17 /18–24 /25–44 /≥45 years) and tobacco use (never user/user). Q1–SES excluded; Q2–age group excluded; Q3–gender/SES excluded; Q4–gender/SES/tobacco use excluded; Q5–SES/tobacco use/age group excluded; Q6, 7, 8–age group and tobacco use excluded. Bold numbers indicate significance (p<0.05).

Overall support was significantly higher for category C packs compared with D by 11% (Table 2) and in Telangana by 49% (Table 4). In Delhi, support was higher for category D compared with C by a significant 23% difference (Table 3).

When category A packs were compared with category B packs, the percentage differences in the choice of packs were not significantly different for most outcomes except that the participants, overall, and in both states, perceived that category A packs would be more likely to lure adults as well as children and adolescents into using tobacco products than category B packs (p<0.05 for both outcomes, overall and in both states). Across all outcomes, the participants perceived category A packs to be significantly less effective than category C and category D packs.

### Perceptions related to the four pack categories among sub-groups

The Supplementary file (S) summarises responses (frequencies) related to perceptions about the four pack categories in the two study areas and the association of perceptions in relation to age groups, area, gender, SES, and tobacco use status.

The 85% GHWs on conventional packs (C) and plain packs (D) were perceived to be most effective in motivating tobacco users to quit, preventing initiation by non-users and most likely to make people think of the health risks of tobacco use by over 90% of respondents. Over 90% of participants also perceived that GHWs were most noticeable and the health warning was easiest to understand on packs C and D (Supplementary Table S1). Nearly 90% of participants believed that packs A and B were most likely and pack D was least likely to lure adults as well as adolescents into using tobacco (Supplementary Table S1).

In Delhi, a higher proportion of participants from both age groups perceived that the GHWs on category D packs were most noticeable, while in Telangana more adolescents perceived that category D pack warnings were more noticeable while the adult group perceived that pack C warnings were more noticeable (p<0.001) (Supplementary Table S2). In Telangana, more participants from the higher SES group perceived that GHWs on packs D (52.5%) than on packs C (47.5%) were more noticeable (Supplementary Table S6).

Category A, the 40% old GHWs on conventional packs, was perceived to be most likely to attract adults (55% in Delhi, 87% in Telangana) and adolescents (59% in Delhi, 81% in Telangana) to use tobacco (Supplementary Table S1). A higher percentage of rural (>80%) than urban (>60%) participants perceived that packs A would be most likely to lure children and adults into using tobacco (Supplementary Table S3). In Telangana, more males (91%) than females (83%) perceived that packs A would be most likely to lure adults while more females (64%) than males (53%) perceived that they would be most likely to lure children and adolescents into using tobacco (Supplementary Table S4). In response to the question ‘the GoI plans to increase the GHW from 40% to 85% which pack would you support?’, 58% of participants in Delhi supported packs D compared to 74% in Telangana who supported packs C (Supplementary Table S1).

## DISCUSSION

Research evidence suggests that plain packs with larger GHWs are effective in reducing pack appeal, increase noticeability of GHWs, have strong public support, have the potential to increase quitting and prevent initiation^25-27^.

Our study found that increasing the size of GHWs to 85% and placing them on plain packaging significantly increased the perceived noticeability of the warnings by over 40% compared with 40% size GHWs on conventional packs, when these packs were shown to the participants at the same time. While there were some variations about whether packs C or D are preferable, the study shows that increasing the size of GHW from 40% to 85% has a negative perceived effect on attracting adults and adolescents to using tobacco. It also has a positive perceived effect on motivation to quit, prevention of tobacco initiation, the seriousness of the health risks of tobacco, and understanding of the message to be conveyed by the GHWs.

This is in keeping with evidence from studies conducted in some of the high-income countries on the perceived effects of plain packaging with large GHWs on tobacco-related outcomes. A study on cigarette pack design with adult French smokers and non-smokers showed that plain packs were perceived to be more effective in convincing smokers to quit and convincing non-smokers not to initiate, compared to other branded packs^28^. Studies conducted in Australia^26^, New Zealand^29,30^ and the UK^31^ suggest that plain packaging with large GHWs reduces smoking appeal, has high public support, promotes cessation and increases attention paid to GHWs as well as recall.

The evidence so far is mainly from high-income countries and may not be generalizable to India. There is limited research on perceptions of the effects of plain packaging from LMICs^32^. This study from India assesses adult and adolescent perceptions of the effects of larger GHWs and plain packaging on noticeability of GHWs, the seriousness of health risks, motivation to quit, and prevention of initiation. Our results mirror findings of studies from high-income countries, thus showing that the results are pertinent to LMIC settings such as India, where tobacco use has been culturally unacceptable.

The study also suggests that SES, gender, age groups, the area of residence or tobacco use status did not influence the perceptions, as unadjusted and adjusted results for regression models were mostly consistent. Across states, there was evidence that all participants of Delhi supported large GHWs on plain packs while only the adolescents from Telangana believed that large GHWs on plain packs would be most effective. Participants from Delhi were primarily residents of urban settings (87.5%) while those from Telangana were primarily residents of rural settings (66%). Possibly, the adolescents in rural Telangana are being taught about harms of tobacco and tobacco control policies in schools and are more receptive to anti-tobacco messaging, reflected in their perceptions. Our findings are in line with those of studies conducted with adolescents in Australia that suggest that plain packs with large GHWs not only reduce the appeal of the pack^9,33^ but also increase their awareness of the health consequences of tobacco use^27^. As adolescents represent a vulnerable target group for experimentation and initiation of tobacco use, and plain packs with large GHWs positively influence adolescents’ perceptions of tobacco use prevention, as observed in our study as well as in previous studies^27,33,34^, it is important that this evidence-based tobacco control measure be implemented in India to protect adolescents from tobacco initiation.

### Strengths and limitations

This Indian cross-sectional study, conducted in Delhi and Telangana, included men and women, urban and rural populations, and adolescent and adult age groups. This study has used well-established sampling methods, validated outcome indicators and designed tobacco packs to assess perceptions of different sizes of GHWs on conventional and plain tobacco packaging.

There are some limitations to this study. We assessed participant perceptions of the effect on outcomes. Hence, we are unable to comment on the actual effectiveness or impact of plain packaging with large GHWs on tobacco initiation, cessation, or other outcomes studied. Further, we used only four types of packs (Figure 1), which represented most likely combinations with respect to pack warnings and types relevant in the Indian policy context. These packs were shown to the participants at the same time to elicit their responses. Hence, our findings are only applicable to these four most likely combinations of packs and warnings, when shown to the participants at the same time.

Earlier studies, mostly conducted in high-income countries such as Australia^21^ and Canada^22,23^ have used smaller samples and Likert scales to assess participant perceptions. However, our community-based study was conducted with a larger sample of 2121 participants, which included nearly 40% participants from rural Indian settings, 50% participants of low socioeconomic status, 21% illiterate population, and 12% children. There is evidence in the literature indicating that Likert scales can pose problems in low literacy populations^35,36^. Hence, we tried to keep the questionnaire simple for these population groups by avoiding Likert scales and asking the participants to directly choose the pack category of their choice for each question. Although the sample size was large, adolescent participants, who are most vulnerable to tobacco experimentation and initiation, were underrepresented. The majority (72%) of respondents had never used tobacco, consistent with the national prevalence rate. Half of the participants were classified as lower or upper-lower and only 4% as upper on the SES scale. While this could have a bearing on our results, evidence suggests that the use of most tobacco products is higher in the lower SES groups^37^. Further, the degree to which participants in Delhi (primarily urban) and Telangana (primarily rural) are aware of the harms of tobacco and tobacco control policies is expected to vary greatly. These differences may have been reflected in our findings.

### Policy implications

This study provides research evidence in favour of larger GHWs, which is of importance in defending the tobacco industry’s legal challenges against the 85% GHWs. Another publication from our group presents findings from a policy analysis in India that emphasizes that the logical next step for India after implementing 85% GHWs is plain packaging^38^. Larger GHWs would contribute towards strengthening implementation of the WHO Framework Convention on Tobacco Control, recommended by the UN for the achievement of Sustainable Development Goal 3 target — reducing premature mortality due to non-communicable diseases by one-third by 2030.

## CONCLUSIONS

When four most likely combinations of pack warnings (40% size GHW, 85% size GHW) and pack types (conventional tobacco packs, plain packs) relevant in the Indian tobacco control policy context in the year 2016 were shown at the same time to adolescent and adult participants from two states of India, 85% size GHWs were perceived to be more effective in increasing noticeability of warnings, motivating cessation, preventing initiation, and conveying the intended health message. Support for plain packaging was higher in Delhi and among adolescents in Telangana. Evidence-based tobacco control measures such as plain tobacco packs with large GHWs are vital to prevent experimentation and tobacco use initiation among vulnerable adolescents in LMICs such as India. Future research should explore more nuanced features of plain tobacco packs focusing on design, placement, and size of text; also field-testing of images depicting different tobacco-attributable health conditions to be used in future rotations of GHWs, as the current GHWs in India are primarily cancer-focused.
